# Perioperative management of patients with asthma during elective surgery: A systematic review

**DOI:** 10.1016/j.amsu.2021.102874

**Published:** 2021-09-20

**Authors:** Samuel Debas Bayable, Debas Yaregal Melesse, Girmay Fitiwi Lema, Seid Adem Ahmed

**Affiliations:** a Department of Anesthesia, College of Medicine and Health Sciences, Debre Berhan University, Debre Berhan, Ethiopia; b Department of Anesthesia, College of Medicine and Health Sciences, University of Gondar, Gondar, Ethiopia

**Keywords:** General anesthesia, Perioperative respiratory adverse events, Endotracheal intubation, Bronchospasm, Laryngospasm

## Abstract

Asthma is one of the commonest respiratory illnesses among elderly patients undergoing surgery. Detailed preoperative assessment, pharmacotherapy and safe anaesthetic measures throughout perioperative period are the keys to decrease complications. Resistance to expiratory airflow results in positive alveolar pressures at the end of expiration, which causes air-trapping and hyperinflation of the lungs and thorax, increased work of breathing, and alteration of respiratory muscle function. This systematic review was conducted according to the Preferred Reporting Items for systematic review and metanalysis (PRISMA) statement. Search engines like PubMed through HINARI, Cochrane database and Google Scholars were used to find evidences. Low-dose IV ketamine, midazolam, IV lidocaine or combined with salbutamol are recommended to be used as premedication before induction. Propofol, ketamine, halothane, isoflurane and sevoflurane are best induction agents and maintenance for asthmatic surgical patients respectively. Among the muscle relaxants, vecuronium is safe for use in asthmatics. In addition, Succinylcholine and pancronium which releases low levels of histamine has been used safely in asthmatics with little morbidity.

## Highlights

• Asthma is one of the commonest respiratory illnesses among patients undergoing surgery.
• This systematic review was conducted according to PRISMA protocol.
• Propofol, ketamine, halothane, isoflurane and sevoflurane are best pharmacologic agents for asthmatic surgical patients.
• Vecuronium is safe for use in asthmatic surgical patients.

## 1 Background

Asthma is defined as a disorder of variable intensity pulmonary disease characterized by airway inflammation and hyper-responsiveness resulting in episodic wheezing, coughing, breathlessness, chest tightness, and reversible airflow obstruction [[1], [2], [3]].

The pathophysiological of asthma is a reduction in airway diameter due to the contraction of smooth muscle, edema of the bronchial wall, and tenacious secretions results airflow obstruction, changes in lung volume, peak flow rate, ventilation-perfusion, altered cardiovascular function and these changes vary in magnitude according to the severity [1,4].

Over 300 million of people worldwide are affected with asthma and Many of them require some type of surgical procedure which needs their asthma should be optimized [5]. Studies showed that prevalence rate and severity of asthma increasing worldwide with variations among different countries ranging from 0.7% to 18.4% [2,3].

Regardless of the techniques of anesthesia, perioperative respiratory adverse events like bronchospasm, laryngospasm, desaturation, coughing and excessive secretion may occur at any stages of the anaesthetic course [1,5].

Surgical patients with history of uncontrolled asthma three months before surgery had nearly double risk of postoperative mortality and three times risk of developing post-operative pneumonia as compared to surgical patients with controlled asthma [6].

To minimize the risk of perioperative respiratory adverse events in asthmatic surgical patients, adequate preoperative assessment and optimization that includes detailed history, pulmonary function test, and medications are imperative. Adequate depth of Anesthesia, using less histamine releasing agents during intraoperative period and close follow up of post op respirator system is a prerequisites to minimize morbidity and mortality among the surgical patients [4].

In many asthmatic patients, treatment with systemic corticosteroids and bronchodilators is indicated to prevent the inflammation and bronchoconstriction associated with endotracheal intubation [7].

### 1.1 Pathophysiology of bronchial asthma

The pathophysiological hallmark of asthma is a reduction in airway diameter due to the contraction of smooth muscle, vascular congestion, edema of the bronchial wall, and tenacious secretions [5]. The airway remodeling; epithelial shedding, subepithelial fibrosis, increased numbers and volume of mucous cells in the epithelium, airway smooth muscle hyperplasia and hypertrophy, and increased vascularization of the airway wall [8]**.** These changes in the extracellular matrix, smooth muscle, and mucous glands causes a decrease in forced expiratory volume in one second (FEV1) and bronchial hyper-responsiveness [9].

Resistance to expiratory airflow results in positive alveolar pressures at the end of expiration, which causes air-trapping and hyperinflation of the lungs and thorax, increased work of breathing, and alteration of respiratory muscle function. In addition, airflow obstruction is not uniform, and the mismatching of ventilation to perfusion occurs, leading to changes in arterial blood gases [8,9].

### 1.2 Justification

Asthma is a common disorder with increasing prevalence rates and severity worldwide. Asthmatic patients often present for surgery and anesthesia and can pose challenges for the anesthetist, especially when General anesthesia with endotracheal intubation is required. A rational choice of anaesthetic agents and airway management based on the available resources in limited resource settings are crucial to minimize perioperative respiratory adverse effects.

This evidence based guideline systematic review focused on comprehensive assessment, risk stratification and identification of uncontrolled asthma, optimization during preoperative period with available drugs. This review suggested also the best possible pharmacologic and anaesthetic techniques. In addition, it provides comprehensive intraoperative and post-operative management algorithms.

## 2 Methods

**Fig. 1:**
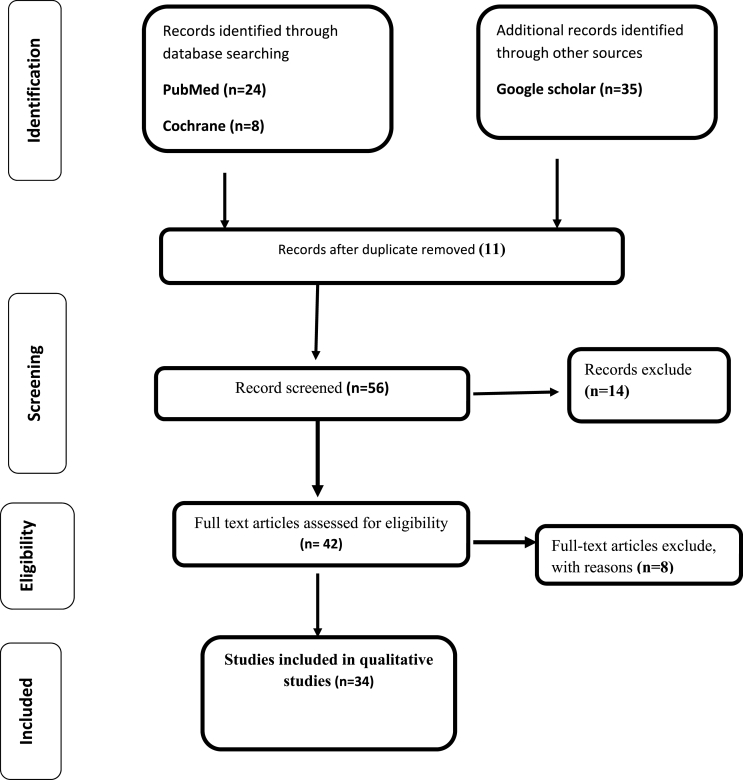
Flowchart for selection of studies by PRISMA flow diagram.

This study was carried out in accordance with the Preferred Reporting Items for Systematic Reviews and Meta-Analyses (PRISMA) 2020 statement [10] (Fig. 1). A computerized systemic research of the PubMed, Google Scholar, and ScienceDirect databases were used to find articles. Prospective observational, interventional studies, meta-analysis, systematic review and audit studies were included in the review using the following MeSH terms: (**Asthma OR Bronchial asthma**) AND (**Surgery OR Elective surgery**) AND (**Anesthesia OR General Anesthesia OR Regional anesthesia**) AND (**Respiratory drugs OR Bronchodilators**) AND **perioperative respiratory adverse events (PRAEs).** In this review, studies on patients with younger than 18 were not included. Publication dates were not used as inclusion or exclusion criteria and only those articles written in English language were considered for this review. Furthermore, after comprehensive and in-depth appraisal of literature, evaluation of quality was conducted according to the According to WHO 2011 level of evidence and degree of recommendation (Table 1).

**Table 1:** Level of evidence and degree of recommendation.

Level	Types of evidence	Degree of recommendation
1a	Meta analyses, systematic reviews of RCTs/guideline/Cochrane systematic review	Strongly recommended/directly applicable
1b	Systematic review with at least one RCTs	Highly recommended/directly applicable
1c	RCTs	Recommended/applicable
2a	Systematic reviews of case control or cohort studies	Extrapolated evidence fromother studies

### 2.1 Selection of studies

Papers fulfilling the following criteria were included in the study: studies presented as original articles, comparative studies on airway intervention for a patient with asthma undergoing surgery, premedication for a patient with asthma undergoing elective surgery, incidence and management of perioperative respiratory adverse events in patient with asthma undergoing surgery, studies written in English.

Studies done on patient with asthma at emergency department, surgery undergoing under local infiltration, child with asthma, studies where full articles were no longer available online were not selected to be included in the current review. All of the research articles that were identified from searches of the electronic databases were imported into the ENDNOTE software version X6 (Tomson Reuters, USA) and duplicates were removed. Before findings had begun, full length articles of the selected studies were read to confirm for fulfilling the inclusion criteria.

## 3 Discussion

The chronic inflammatory process leads to tissue injury and subsequent remodeling of the airway structure. Bronchospasm and mucous plugging obstruct both inspiratory and expiratory airflow. Optimization of patients with asthma at preoperative, intraoperative and postoperative period play a pivotal role for a good out come of the victims.

### 3.1 Preoperative assessment and management

#### 3.1.1 Preoperative assessment

Most well-controlled asthmatics tolerate anesthesia and surgery well. The incidence of perioperative bronchospasm in asthmatic patients undergoing routine surgery is less than 2%, especially if routine medication is continued. However, perioperative respiratory adverse events are increased in patients over 50 years undergoing major surgery and in those patients with unstable disease.

Poorly controlled asthmatics patients with current symptoms, history of frequent exacerbations or hospital admissions are at risk of perioperative respiratory problems; bronchospasm, sputum retention, atelectasis, infection and respiratory failure. Hence, to minimize perioperative respiratory adverse events elective surgery should take place when the patient's asthma is optimally controlled [11]**1b**.

The purpose of preoperative evaluation in asthmatic patients is used to determine the respiratory dysfunction and its magnitude, the effectiveness of current therapy and prepare suited anaesthetic plan [2]**1b.** Preoperative assessment should focus and include: Activities of daily living (ADL) and physical status, presence of infectious symptoms, amount and purulence of sputum, presence of allergies, Factors known to trigger attacks or exacerbations, use and effectiveness of medications, previous history of surgery and anesthesia, coexisting medical disorders and obesity or sleep apnea syndrome [11].

History of asthma-specifically emergency visits, hospitalizations, ICU stay, and use of systemic corticosteroids are independent contributing factors for postoperative major adverse outcomes in asthmatic patients and should be addressed preoperatively by the anesthetist [6]1b. Preoperative examination before the induction of anesthesia should include observation of respiratory rate and auscultation of both lung fields (presence or absence of adventitious lung sounds). Asthma criticality assessment [12]**2a** (Table 2).

• Physical examinations

**Table 2:** Assessment of severity of asthma.

Clinical assessment	Well controlled	Not well controlled	Poorly controlled
1. Symptoms (wheezing, shortness of breath, chest tightness)	≤2 days/week	>2 days/week	Daily
2. Night time awakenings with breathing problems	≤2 x/month	3–4 x/month	>1 x/week
Short-acting beta 2 agonist use for rescue	≤2 days/week	>2 days/week but not daily	Daily
Interference with normal activity	None	Some limitation	Extreme limitation
Exacerbations requiring systemic corticosteroids	≤1 x/year	2–3 x/year	>3 x/year

Patients above 5 years include additionally			
FEV1 predicted	>80%	60–80%	<60%
FEV1/FVC	>0.8	0.75–0.80	<0.75

Anesthetists should focus on detecting signs of acute bronchospasm or active lung infection, chronic lung disease and right heart failure. When expiratory airflow is markedly decreased, breath sounds are diminished or inaudible. A simple screening test for prolonged exhalation is the forced expiratory time (FET), which can be assessed by listening over the trachea while the patient exhales forcibly and fully. An FET >6 s correlates with a substantially lowered FEV1/FVC ratio and should initiate further investigation [5]1b. Preoperative wheezing is predictive of a difficult perioperative course.

• Investigations

Even though, investigation for asthmatic patients is not routine done in low resource setting areas, the below mention laboratory investigation may help the perioperative team for decision on the severity of asthma and to predict post-operative respiratory adverse events.

1. **Pulmonary function test/Spirometry:** this investigation helps for chronic and uncontrolled asthmatic patients to determine whether airflow obstruction is at least partially reversible after use of bronchodilators in patients of all ages, reversibility is indicated by an increase of at least 12% in FEV1 from baseline. In adults, an increase in FEV1 of greater than 200 mL from baseline also constitutes reversibility [12,13]**2a and 1b** respectively.
2. **Electrocardiogram (ECG):** asthmatic patients may show right atrial or ventricular hypertrophy, acute strain, right axis deviation, and right bundle branch block, so we should routinely obtain an ECG during and after the acute attack for comparison.
3. **Chest x-rays:** is useful to rule out pneumonia or heart failure, Hyperinflation and increased lung markings, bronchial thickening are common radiographic findings of asthma but has little value in formulating changes in therapy [2]1a.

#### 3.1.2 Preoperative pharmacologic optimization of asthma

A stepwise approach to managing asthma, such as the treatment regimen proposed by the Global Initiative for Asthma [14]**1b**, is recommended to maintain preoperative disease control in asthmatics scheduled to undergoing surgery under general anesthesia [15]1b. Patients are evaluated and placed on a discrete treatment “step” based upon symptoms and severity of disease. As the disease increases in severity, the number and types of medications used to treat the patient also increase. This model of increasing therapy based on symptom control is easily applied to preoperative preparation of asthmatics [2]**1b** (Table 3).

**Table 3:** Stepwise approach to the preoperative treatment of asthmatic patients based on their degree of asthma control.

Severity of Asthma	Symptoms/characteristics	Pharmacologic intervention
Well controlled asthma	o No currents symptoms o No symptoms in the past 6 months. o No history of taking any drugs in the past 6 months.	⁃ Continue without medication.
Controlled asthma	o No current symptoms o No change in symptoms for previous 6 months. o Hx of taking inhaled corticosteroid (ICS) o Use of long acting beta 2-agonists (LABA)	⁃ Continue ICS ⁃ LABA
Bronchial asthma with recent changes	o Recent change of symptoms after admission for surgery. o Hx of using rescue short acting beta blockers (SABA). o No history of taking oral corticosteroids	⁃ Start ICS ⁃ Consider LABA ⁃ SABA preoperatively if no LABA. ⁃ OCS for 3–5 days preoperatively.
Moderate bronchial asthma	o Daily symptoms of bronchial asthma. o Current Taking of LABA. o Occupational use of OCS. o Current taking of ICS.	⁃ Continue ICS ⁃ Continue LABA. ⁃ SABA preoperatively if no LABA ⁃ OCS for 3–5 days preoperatively
Severe bronchial asthma	o Daily and sever symptoms of asthma. o Use of ICS o Use of LABA o Daily use of OCS.	⁃ Continue ICS. ⁃ Continue LABA. ⁃ SABA preoperatively if no LABA. ⁃ Increased dose of OCS for 3–5 days preoperatively.

ICS: Inhaled corticosteroids, LABA: Long acting Beta 2-agonists; OCS: oral corticosteroids; SABA: short acting Beta 2-agonists.

Inhaled corticosteroids like beclomethasone (40 μg 2x/daily) is the cornerstones to stabilize persistent asthma and decrease morbidity and mortality in asthmatic surgical patients [11]1b. in Addition, parenteral steroids such as hydrocortisone (200 mg IV stat) and methyl prednisolone (40–80 mg IV per day) for 5 days remain a mainstay of the treatment of acute exacerbation of asthma [11]1b. Patient with history of using long-acting β2 agonists could be associated with a clinically significant number of unnecessary hospitalizations, intensive care unit admissions, and deaths each year [11]1b.

Combined treatment with corticosteroids and a β2-adrenergic agonist (methylprednisolone 40 mg/day orally) and salbutamol respectively can improve preoperative lung function and decrease the incidence of wheezing following endotracheal intubation [4]1b. In smoker patients with uncontrolled asthma for elective surgery should stop smoking at least 6–8weeks before surgery to allow the greatest recovery of endobronchial cilia mucus clearance [16]1a.

Patients who are on systemic corticosteroids for >2 weeks during the prior 6 months should be considered at risk for adrenal suppression needs intra operative supplementation of 1–2 mg/kg of hydrocortisone iv every 8 h and more on the day of surgery followed by return to previous dosage by gradual tapering off [1,5]**1b** and **1a** respectively.

According to Enright preoperative management of acute exacerbated asthma should treated with steroids and inhaled beta 2-agonists (Oral methylprednisolone 40 mg for 5 days before surgery) in addition, patients with a preoperative FEV1<80% of predicted should receive steroids (40–60 mg of prednisone/day or hydrocortisone 100 mg *IV* TID), Infections should be eradicated and Fluid and electrolyte imbalances should be corrected [17]**1a.**

### 3.2 Premedication

An optimal premedication that alleviate anxiety, favored sympatholsis and antisialogue effects may improve work of breathing and possibly avert bronchospasm during induction. However, there is no ideal drug, which can favor the above mention goals without side effects in low resource settings like Ethiopia. Administration of 0.5 mg/kg Midazolam for anxious patient is safe and effective to alleviate anxiety and anxiety induced bronchospasm [18]**1a.**

A prospective randomized double blinded study showed that administration of 1.5mg/kg- 2 mg/kg IV lidocaine 90 s before laryngoscopy effectively suppress the cough reflex and attenuates increases in HR and MAP [19]**1a.**

Opioids are not recommended for asthmatic patients. However, combination of low dose ketamine (0.15 mg/kg IV) with that of (2 mcg/kg IV) fentanyl effectively provide analgesia and prevents fentanyl induced cough [20]**1a** (Table 4).

**Table 4:** Summary on perioperative anaesthetic management of asthma.

Premedication	Midazolam (Oral/IV)	0.5 mg/kg oral or IV to alleviate anxiety
	Lidocaine (IV)	1.5–2 mg/kg IV lidocaine 90 s before laryngoscopy suppress the cough reflex and attenuates increases in HR and MAP
	Salbutamol (inhalational)	Salbutamol puff 5–10 min before induction.
Preemptive analgesia	Ketamine	0.15 mg/kg IV for analgesia and prevent fentanyl induced cough.
	Fentanyl	1-2mcg/kg Iv for analgesia
Anaesthetic agents(IV)	Propofol	Recommended in thermodynamically stable patient
	Ketamine	Recommended in thermodynamically unstable patients
Volatile anesthetics	Sevoflurane and Halothane	Depresses airway reflexes and produce direct bronchial smooth muscle relaxation.
	Desflurane and isoflurane	irritant to airway apparatus and increases airway resistance
Muscle relaxants and reversal agents	Suxamethonium	Choice for rapid sequence induction.
	Vecuronium, Pancuronium	Safe to use in asthmatic patients
	Neostigmine	Safe to use as reversal agents.
Airway management	• Warm, humidified gases should be provided at all times. • If the surgery allowed use noninvasive airway intervention, Face **mask > LMA > ETT.** • Optimal depth of anesthesia	
Extubation	• Adequate suctioning under optimal depth of anesthesia. • Deep and smooth extubation is recommended if difficult intubation were not encountered during induction.	
Post-operative	• Immediate oxygen supplementation and CPAP. • Meticulous monitoring of SPO2, Oxygen supplementation via nasal prongs. • Adequate hydration and analgesia.	

### 3.3 Induction and intraoperative management

Adequate depth of anesthesia is required to prevent bronchospasm and reduce the response to tracheal intubation. Severe bronchospasm may cause fatal or near-fatal events such as irreversible brain damage due to inability to ventilate. Deep level of anesthesia can be achieved through a combination of appropriate IV anaesthetic agents, ultra short acting opioids and volatile agents prior to instrumenting the airway, as tracheal intubation during light levels of anesthesia can precipitate bronchospasm [1].

Among the intravenous anaesthetic agents propofol and ketamine have bronchodilation effect. Propofol is the induction agent of choice in the hermodynamically stable patient due to its ability to attenuate the bronchospastic response to intubation both in asthmatics and non-asthmatics [[21], [22], [23]]1b. A systematic review showed that Ketamine is an ideal induction agent for hermodynamically unstable asthmatics patients due to its ability to produce direct smooth muscle relaxation and bronchodilation without decreasing arterial pressure or systemic vascular resistance [24,25]1a.

Studies indicated that volatile anesthetics especially halothane, isoflurane and Sevoflurane are excellent choices for general anesthesia, as they depress airway reflexes and produce direct bronchial smooth muscle relaxation [26,27]2a. However, desflurane which causes irritant to airway apparatus and increases airway resistance should be avoided in asthmatics [[28], [29], [30]]1b (Table 4).

### 3.4 Muscle relaxants

Generally, neuromuscular blocking agents are the most common medications to cause allergic reactions in the operating theatre [31]1c. Even though, suxamethonium can releases low levels of histamine, it has a great useful for the asthmatic that needs a rapid sequence induction in low resource settings without significant morbidity and mortality [1]. A systematic review supported that Vecuronium, rocuronium, and *cis*-atracurium are safe for use in asthmatics during induction and maintenance while pancronium which releases low levels of histamine, has been used safely in asthmatics with little morbidity [27].

### 3.5 Airway management

Warm, humidified gases should be provided at all times. Rapid sequence or standard induction should be performed as indicated as long as adequate anesthesia is assured; succinylcholine is not contraindicated for rapid sequence induction. The decision whether to intubate the trachea, provide anesthesia by mask, or use a laryngeal mask airway (LMA) is based on the type of surgery, patient condition and other clinical parameters. However, there is evidence that tracheal intubation causes reversible increases in airway resistance not observed with placement of LMA [5,32]2a. Deep and smooth extubation is recommended if airway difficulties were not encountered during induction but when difficulty is anticipated the patient may be taken to the post anesthesia care unit as intubated with opioids administered to facilitate tolerance to the endotracheal tube. When the patient is awake and possesses appropriate airway reflexes, Extubate with IV lidocaine to prevent bronchospasm [5]**2a**.

Inadequate depth of anesthesia at any point can allow bronchospasm to be precipitated. Anaesthetic maintenance with a volatile agent such as isoflurane or sevoflurane confers protective bronchodilation. In selecting a ventilatory mode, attention should be given to providing an adequately long expiratory time to avoid the build-up of intrinsic or auto-PEEP. This can be facilitated by using smaller tidal volumes than usual [1]**2a**.

### 3.6 Post-operative management

Intraoperative course is a major determinant for post-operative management, if the surgery was uneventful and pain, nausea, and respiratory status are well-controlled, asthmatics may safely be discharged to the appropriate inpatient unit without further intervention, but in significant intraoperative complications with severe bronchospasm, special care must be taken to ensure patient safety during the postoperative period [11] **1b**.

Postoperative control of the amount of sputum, recovery and maintenance of ventilator gas exchange become possible with early respiratory rehabilitation, leading to prevention of complications and early discharge from hospital [6]**1c**. Respiratory rehabilitation should be performed by a team of physicians, nurses, physiotherapists together with the patient's family, if necessary [16]**1a**.

It is prudent to re administer β-agonists prior to emergence and throughout the postoperative recovery period as needed for recurrent bronchospasm and maintaining a head of the bed up position is preferable for prevention of atelectasis [33]**1b**. Ensure patient's usual medications are prescribed in an appropriate formulation after surgery [34]**1b**. So that, prescribe Salbutamol regular, Review dose and route of administration of steroid daily and Avoid NSAID in poorly controlled asthmatics [34]**1b** is paramount during the post-operative period.

## 4 Conclusion

In many asthmatic patients, treatment with systemic corticosteroids and bronchodilators is indicated to prevent the inflammation and bronchoconstriction associated with endotracheal intubation.

Low-dose ketamine (0.15 mg/kg IV, midazolam 0.5 mg/kg), Intravenous lidocaine (1–1.5 mg/kg) or combined with salbutamol, Standard dose of Anti muscarinic are safe drugs used as premedication before induction.

Propofol, ketamine, halothane, isoflurane, sevoflurane are best induction agents and maintenance for asthmatic surgical patients. In addition, vecuronium is safe for use in asthmatics during induction and maintenance, Succinylcholine and pancronium which releases low levels of histamine, has been used safely in asthmatics with little morbidity, while atracurium and mivacurium should avoid as much as possible.

Even if some literatures are in controversial most of them agreed on deep level of intubation and extubation for asthmatic patients. According to high level controlled studies the perioperative complication from low to high in asthmatic patients **Regional < Facemask < LMA < ETT.**

**Fig. 2:**
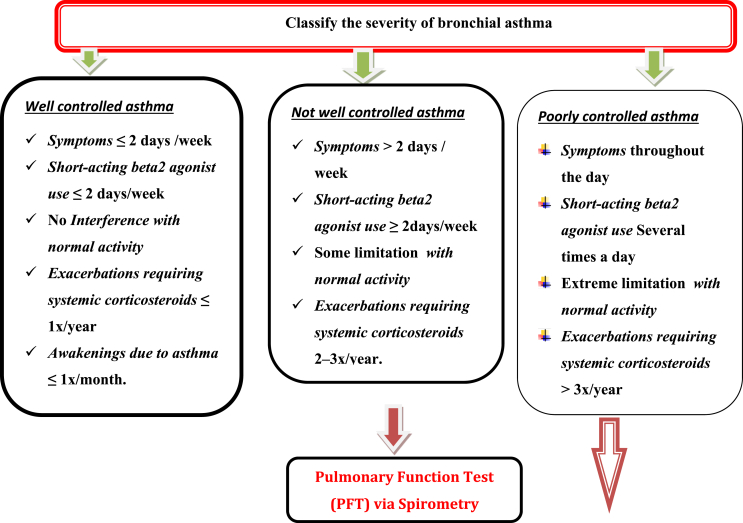

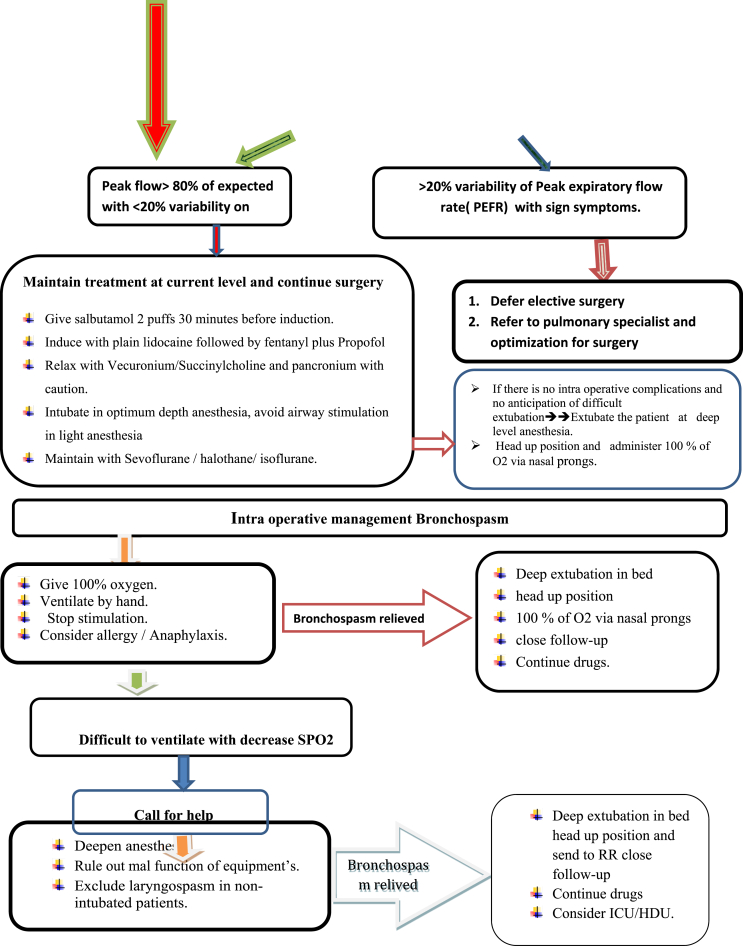

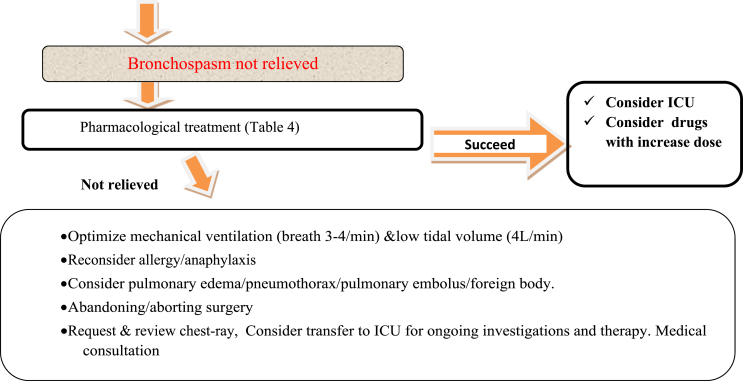
Flow diagram of Perioperative management of patients with Asthma during elective surgery.

Reversals such as neostigmine (40 μg/kg) with atropine (10 μg/kg) mixture could safely be used for patients with airway hyperactivity. The Post-operative management mainly depends up on the Intraoperative course, if surgery was uneventful, and pain, nausea, and respiratory status are well-controlled, asthmatics may safely be discharged either to home or to an appropriate inpatient unit without further intervention, but in significant intraoperative complications with severe bronchospasm, special care must be taken to ensure patient safety during the postoperative period (Fig. 2).

## Registration of research studies

Name of the registry: researchregistry

Unique Identifying number or registration ID: reviewregistry1215.

Hyperlink to your specific registration (must be publicly accessible and will be checked): https://www.researchregistry.com/browse-the registry#registryofsystematicreviewsmeta-analyses/.
